# CMR of microvascular obstruction and hemorrhage in myocardial infarction

**DOI:** 10.1186/1532-429X-14-68

**Published:** 2012-09-29

**Authors:** Katherine C Wu

**Affiliations:** 1 Division of Cardiology, Department of Medicine, Johns Hopkins Medical Institutions, 600 N. Wolfe Street/Carnegie 568, Baltimore, MD, 21287, USA

**Keywords:** Microvascular obstruction, Myocardial infarction, Cardiac magnetic resonance, Myocardial hemorrhage

## Abstract

Microvascular obstruction (MO) or no-reflow phenomenon is an established complication of coronary reperfusion therapy for acute myocardial infarction. It is increasingly recognized as a poor prognostic indicator and marker of subsequent adverse LV remodeling. Although MO can be assessed using various imaging modalities including electrocardiography, myocardial contrast echocardiography, nuclear scintigraphy, and coronary angiography, evaluation by cardiovascular magnetic resonance (CMR) is particularly useful in enhancing its detection, diagnosis, and quantification, as well as following its subsequent effects on infarct evolution and healing. MO assessment has become a routine component of the CMR evaluation of acute myocardial infarction and will increasingly play a role in clinical trials of adjunctive reperfusion agents and strategies. This review will summarize the pathophysiology of MO, current CMR approaches to diagnosis, clinical implications, and future directions needed for improving our understanding of this common clinical problem.

## Review

### Introduction

Microvascular obstruction (MO) occurs in the setting of reperfusion following prolonged myocardial ischemia and provides incremental prognostic information beyond infarct size, to which it is related. MO is characterized by a number of ultrastructural and functional changes at the microvascular level. Understanding these histopathophysiologic changes can inform the approach by cardiovascular magnetic resonance (CMR) to detecting MO and our interpretation of the results and their subsequent clinical implications. It can also help potentially improve how MO is assessed by CMR which has implications with regard to our understanding of infarct evolution and healing as well as the evaluation of the efficacy and mechanisms of action of adjunctive reperfusion agents. This review will discuss the various pathophysiologic derangements, both anatomic and functional, seen at the microvascular level after myocardial reperfusion; current and evolving CMR techniques to assess MO and the related phenomenon of myocardial hemorrhage; the temporal evolution of MO and its relationship to infarct healing, adverse LV remodeling and clinical prognosis; clinical trial applications; and future directions.

### Pathophysiology of microvascular dysfunction

**Figure 1:**
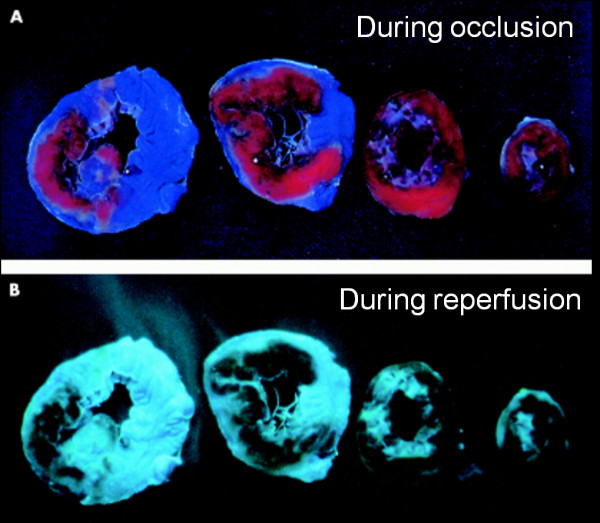
**Shown is an example of anatomic no-reflow detected by vital staining in an experimental rabbit model of coronary occlusion and reperfusion**. In Panel **A**, 4 short-axis slices are shown depicting the regions perfused and stained by monastral blue, which was injected during coronary occlusion. Regions which were not perfused by the dye (i.e. do not stain blue) represent the risk area. In Panel **B**, the slices were imaged under UV light to depict the fluorescent areas (greenish color) stained by thioflavin-S, which was injected following reperfusion and stains intact endothelium. The thioflavin-S negative regions represent no-reflow,are contained within and are smaller than the risk region. Hence, thioflavin-S negative areas depict regions of obstruction to flow despite reperfusion . Reprinted with permission from Reffelmann T, Kloner R A Heart 2002;87:163 [2].

The contribution of microvascular injury in causing anatomic myocardial “no-reflow” was first described in the 1970’s [1-3]. At that time, it had already been recognized in other organs that despite recanalization or restoration of arterial flow to an ischemic tissue bed, perfusion at the tissue level may not uniformly occur. In a canine model of ischemia-reperfusion, Kloner et al. studied the evolution of myocardial injury using a combination of electron microscopy and the vital dye, thioflavin-S, which stains intact endothelium and thus causes myocardial tissue receiving adequate capillary flow to fluoresce under UV light [1]. Epicardial coronary reperfusion after prolonged ischemia resulted in subendocardial regions of nonfluoresence (Figure 1) representing no-flow or low-flow which were confirmed to have substantially reduced regional blood flow measured by quantitative radioactive microsphere analysis [4,5]. Because of the wavefront of necrosis, the endocardium is the most vulnerable area for ischemic damage.

**Figure 2:**
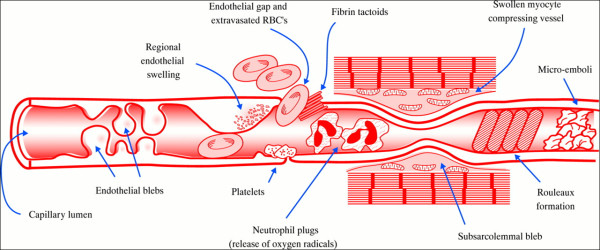
**Schematic depicting the multiple mechanisms that contribute to the no-reflow phenomenon at the ultrastructural level.** Reprinted with permission from Reffelmann T, Kloner R A Heart 2002;87:164 [2].

Under electron microscopy, these areas of nonfluorescence were characterized by striking ultrastructural changes to the microvasculature in addition to evidence of irreversible myocardial cell injury (Figure 2). Under normal circumstances, the capillary lumen is widely patent and lined by intact endothelial cells. In contrast, within the nonfluorescent, thioflavin-negative regions following reperfusion, microvessel integrity was compromised with intraluminal obstruction of the lumen caused by regional swelling of the endothelial wall as well as intraluminal protrusions and blebs of the endothelial cytoplasm. Plugging of the microvessels by erythrocytes, platelets, and neutrophils was also seen. Also contributory was external compression of the capillaries by edematous myocytes and subsarcolemmal blebs. Based on these experimental observations, regions of microvascular injury characterized by the aforementioned ultrastructural anatomic abnormalities and reduced regional blood flow following reperfusion were termed anatomic “no-reflow”. The quantitative extent of the anatomic no-reflow zone is determined by how well the vital staining dye penetrates into the ischemic tissue bed.

Although the exact mechanisms and time course of events leading to anatomic no-reflow are unknown, it is hypothesized that a number of interrelated factors contribute [3,6]. Direct endothelial damage caused by ischemia which leads to the observed protrusions of the endothelial surface that encroach into and compromise the capillary lumen likely play a direct role in the perfusion deficits. Further plugging of the lumen by erythrocytes, fibrin, and platelet thrombi suggest that reperfusion leads to some degree of initial flow into the microvessels which is later compromised. Leukocyte plugging is also observed and this may contribute not only to mechanical obstruction but may also lead to reduced endothelial dependent and independent vasodilation through oxidative stress pathways. Neutrophils elaborate reactive oxygen species that can impair endothelial and platelet function. Oxygen free radicals can also promote the synthesis of tissue factor which may accelerate the accumulation of fibrin/fibrinogen.

In humans, clinical no-reflow is characterized by the additional important contributing factors of microembolization and the resultant inflammatory response [6,7]. Rupture or erosion of epicardial coronary artery plaques, either spontaneously or induced mechanically by percutaneous coronary intervention, can lead to distal showering of embolic debris, consisting of atherosclerotic plaque components and/or thrombotic material. Distal embolization may not only contribute to mechanical obstruction of the microvessels but also causes an inflammatory response with the elaboration of vasogenic and thrombogenic factors that further exacerbate existing microvascular dysfunction. Hence, pathophysiologically, no-reflow is the result of the complex interplay of a number of related processes.

### CMR techniques for microvascular obstruction

**Figure 3:**
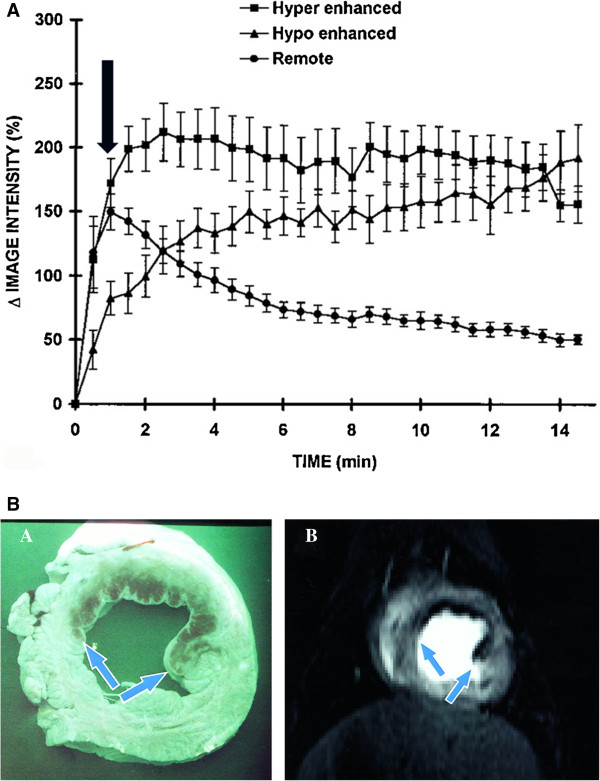
**A shows the signal intensity curves following gadolinium bolus administration in infarct regions which become hyperenhanced compared to the persistent hypoenhancement within the infarct core.** Historically, hypoenhancement was measured at ~1 min (black arrow) following contrast administration. Reprinted from Judd, RM et al. Circulation 1995;92:1902–10 [5]. **B** shows the correlation between MO detected by thioflavin-S (left panel) compared to CMR (right panel). Reprinted from Rochitte C E et al. Circulation 1998;98:1006–1014 [14].

**Figure 4:**
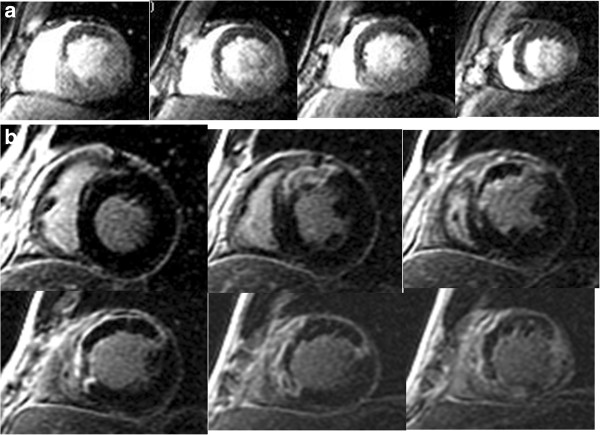
MO regions depicted on first pass imaging CMR (top panel) which though smaller, persist on LGE images (bottom panel).

The advent of fast CMR techniques in the 1990’s facilitated the study of the temporal perfusion patterns within acute reperfused infarcts following bolus administration of gadolinium by allowing a temporal resolution of seconds rather than minutes [8-10]. In seminal publications involving an experimental animal model and a parallel study of patients with acute reperfused infarcts [5,11], regions of myocardial hypoenhancement within the first 2 min of contrast administration were observed within the infarct region that were characterized by significantly reduced regional blood flow and correlated in size with anatomic no-reflow zones measured by thioflavin-S (Figures 3 and 4). Subsequently, these hypoenhanced areas seen on CMR were termed microvascular obstruction (MO) and since they were measured within 1–2 min of contrast administration, represent “early” MO. The extent of early MO by CMR was found to correlate closely with the size of the anatomic no-reflow region by thioflavin (r = 0.91) but was significantly smaller, measuring 61% of the pathologic region [12]. Regions of CMR MO most closely approximated regions within the infarct corresponding to <40% of remote myocardial blood flow at 2–9 days after reperfusion, compared to thioflavin-S negative regions which corresponded to regions with <50% of remote flow [12] . In these original studies of CMR MO, a magnetization-driven spoiled gradient-recalled echo pulse sequence was used that did not require adjustment of the inversion time [13].

**Figure 5:**
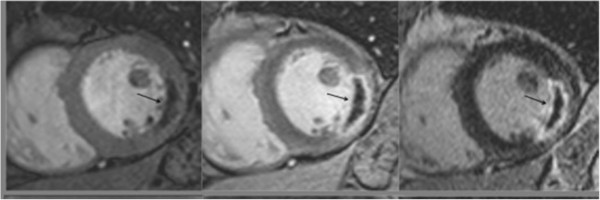
**MO (arrow) within a lateral infarct shown using high-resolution k-t SENSE accelerated first pass imaging (left panel), EGE with fixed TI of 440 msec (middle panel), and LGE (right panel).** Reprinted with permission from Mather et al. J Cardiovasc Magn Reson 2009;11(1):11–33 [21].

**Figure 6:**
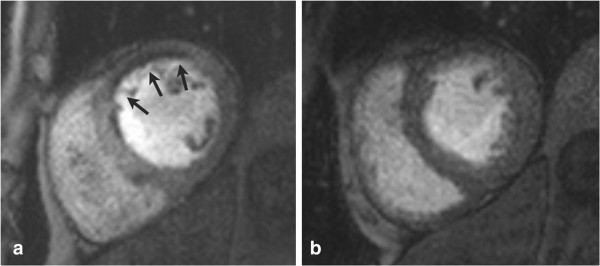
**Shown are corresponding short-axis slices from a 62-year-old gentleman with anterior ST elevation MI who was treated with primary PCI.** Using the kt-SENSE first-pass perfusion imaging technique on day 2 post MI with 0.1 mmol/kg intravenous gadolinium contrast, there is an area of persistent hypoperfusion corresponding to MO. There is substantial improvement at re-imaging on day 7 (Panel **b**). (Image provided courtesy of Ananth Kidambi, BMBCh; Adam N. Mather, MBBS; and Sven Plein, MD, PhD; Multidisciplinary Cardiovascular Research Centre & Leeds Institute of Genetics, Health, and Therapeutics, University of Leeds, Leeds, United Kingdom.).

There have since been numerous additional approaches to assessing CMR MO in the setting of improved pulse sequences. First-pass perfusion sequences tracking the first minute of gadolinium arrival and distribution within the myocardium have been used to detect MO [15-17] (Figure 5). However, these original sequences suffered from relatively low spatial resolution (~2.7 × 3.4 mm) and reduced LV coverage (maximum of 3–4 slices) which reduce diagnostic sensitivity. Subsequently, with the routine use of ultrafast inversion-recovery gradient echo sequences for infarct delineation, MO is increasingly assessed on late gadolinium enhanced (LGE)(Figure 5) as well as early gadolinium enhanced (EGE) images [18-20] with the advantage of improved spatial resolution as well as complete LV coverage. Assessing MO on EGE requires a fixed, high inversion-time (TI) and is generally performed at ~2-4 min following contrast administration (Figure 6). With the recent advent of accelerated high-resolution perfusion sequences (1.5 mm × 1.5 mm spatial resolution, 8 slices acquired over 2 R-R intervals) [21], it is now possible to detect true first pass MO and quantify it for the entire LV (Figure 6).

Because gadolinium is not a pure intravascular agent and extravasates into the interstitium within minutes following administration, it gradually diffuses into the initially hypoenhanced zone. Hence, the size of MO decreases over time from contrast administration, the largest being during first pass and the smallest during LGE (the latter also termed “persistent” MO in the literature). Although there is a high concordance and correlation amongst the 3 measures of MO (r = 0.91 between FP and EGE; r = 0.55-0.78 between EGE and LGE MO) [16,21], MO will be detected more frequently on FP than by EGE and LGE (incidence of 22% by FP vs. 14% by LGE in one study [21]). Amongst the 3 measures, there was the least variability in MO *quantification* using the LGE method [21]. First-pass MO may be overestimated by the concomitant presence of significant epicardial coronary stenoses which impede contrast delivery. Late or persistent MO presence and extent will depend upon the timing of image acquisition following gadolinium administration, particularly in the presence of less severe microvascular dysfunction.

### Time course of MO post-infarction

MO extent varies as a function of time from the acute ischemic event. Experimentally, it has been demonstrated that there is an expansion of the anatomic no-reflow area by thioflavin-S in the hours following reperfusion, with a tripling in size between 2 min and 2–4 h and a further smaller increase up to 8 h following reperfusion [4,22,23]. Following an initial hyperemic phase within the first 2 min of reperfusion, there is a marked, progressive decline in myocardial blood flow which plateaus at around 50% of normal flow, which supports these findings. Experimental studies using CMR, in addition to thioflavin staining and radioactive microspheres to measure blood flow, have also suggested that MO extent increases from 1–2 h to 24 h post-reperfusion and may further increase up to 48 h post-reperfusion [14,24,25]. However, there have not been any confirmatory studies in humans to demonstrate expansion of MO at any timepoint post-reperfusion.

**Figure 7:**
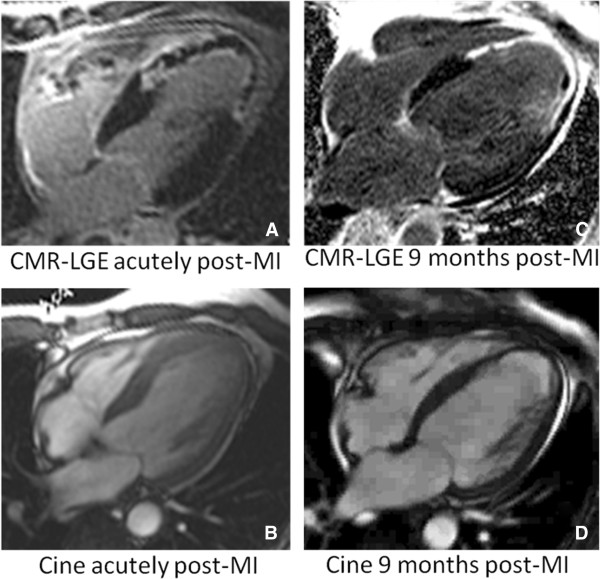
**The effect of MO on adverse LV remodeling is shown in a patient. CMR-LGE and cine images are shown acutely post-infarction (Panels A & B) and at 9 month follow-up post-infarction (Panels C & D).** Despite successful epicardial reperfusion of the left anterior descending coronary artery, there was a large region of persistent MO associated with a large anteroapical infarct measuring 49% of the LV (Panel **A**). Acutely (Panel **B**), LVEF measured 27% and LV volumes were enlarged with LV end-diastolic volume (LVEDV) of 192 ml and end-systolic (LVESV) of 141 ml. At 9 month follow-up, there was LV apical aneurysm formation with infarct wall thinning (Panel **C**) with a persistently reduced LVEF of 25% and further enlargement of the LV with LVEDV of 291 ml and LVESV of 218 ml (Panel **D**).

It is increasingly apparent that the temporal course of MO may vary in the subsequent days to weeks as infarct healing ensues and may in fact influence LV remodeling. Both animal models and humans studies, using both CMR and myocardial contrast echocardiography to assess MO, demonstrate that microvascular dysfunction may persist up to 1 month post-reperfusion [26-28]. Persistence of MO at 1 month was associated with worse regional wall motion, scar thinning, and infarct expansion [26,27]. In a rat model, at 4 weeks post-reperfusion, regions of anatomic no-reflow remain visible and are associated with a markedly reduced capillary density and decreased myocardial blood flow [27]. A recent study using a porcine infarct model lends further insight by showing that histopathologic evidence of obstructed capillaries filled with necrotic debris can be seen at 2 weeks but the infarct is replaced by extensive collagen deposition and fibrosis at 6 weeks [29]. However, particularly in patients, microvascular dysfunction may in fact resolve much earlier than 1 month (Figure 7), as several CMR and myocardial contrast echocardiography studies have shown, and earlier resolution appears to correlate with improved functional recovery post-MI and outcome [28,30-32]. The severity of MO likely affects its degree of persistence and may account for conflicting data regarding temporal changes in the size of MO in experimental studies with some studies showing no significant change in MO extent between 2 and 9–10 days [12,25] and others in which MO appeared smaller at 1 week [29,33]. Also contributory may be the complex pathophysiologic mechanisms of MO that can be differentiated as “structural” versus “functional” [34]. “Structural” or irreversible alterations to the microvascular bed can be characterized by the luminal obstruction by cellular debris and endothelial wall damage. In humans in particular, there may be a larger contribution of “functional” or potentially reversible changes such as microvascular spasm and microembolization which promotes vasoconstriction via the elaboration of vasoactive and proinflammatory mediators [35] and may result in reduced myocardial perfusion reserve which can be potentially assessed by adenosine myocardial perfusion [36,37]. The time courses of structural and functional no-reflow may thus differ and explain in part, variability in the time course data. Functional no-reflow may perhaps resolve more quickly as vasoactive/proinflammatory mediator levels return to normal post-infarction compared to structural no-reflow that requires prolonged infarct tissue healing.

### Impact of MO on clinical outcome and adverse LV remodeling

**Figure 8:**
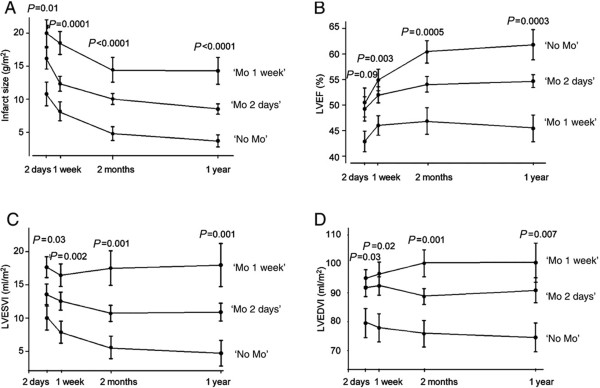
**The presence and persistence of MO can predict how well the LV remodels in the subsequent year.** Reprinted with permission from: Ørn S et al. Eur Heart J 2009;30:1978–1985 [30].

**Figure 9:**
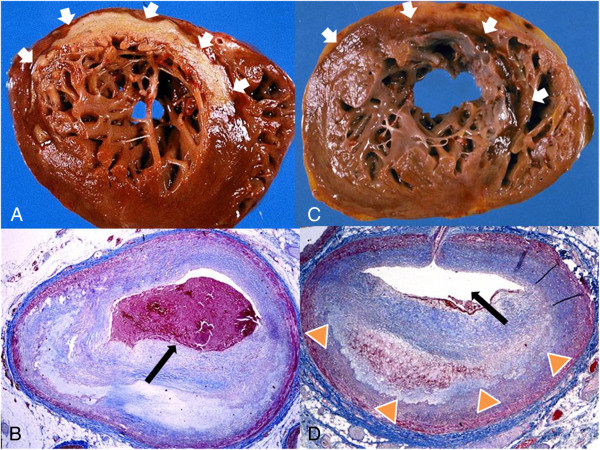
**Gross anatomic features (Panels A & C) of a bland, white non-reperfused (Panel A, white arrows) and a hemorrhagic, red reperfused (Panel C, arrows) acute MI.** Coronary cross sections (Panels **B** &**D**) revealed an occlusive thrombus (Panel **B**, black arrow) in the left anterior descending artery (LAD) of the bland infarct while the hemorrhage infarct was associated with recanalization of the LAD (Panel **D**, black arrow) with residual mural thrombus (Panel **D**, orange arrowheads). Reprinted with permission from Basso C, Thiene G. Heart 2006;92:1559–1562 [62] (Original image provided courtesy of Professor Cristina Basso, Department of Medical Diagnostic Sciences and Special Therapies, University of Padua Medical School, Padova, Italy.).

With the caveat that many of the studies were relatively small, which limits the robustness of multivariate modeling, CMR MO has been found to be predictive of clinical outcome (Table 1), independently of or when adjusted for other indices such as infarct size and LVEF. This is consistent with the results of studies using other imaging technique such as myocardial contrast echocardiography and coronary angiography to assess no-reflow [23,38-42]. Many of these patient outcome studies also showed a relationship between presence of MO and adverse LV remodeling (Table 2) with reduced global systolic function and larger LV volumes at follow-up (Figures 8 and 9), suggesting a possible mechanism for the poor prognosis. Experimental studies have supported these observations. In an animal model, a larger region of anatomic no-reflow was associated with thinner infarct walls and worse infarct expansion [27]. Theoretically, obstruction of blood flow to the infarct core could limit the delivery and transit of cellular components such as macrophages required for phagocytosis of cellular debris and nutrients needed for optimal infarct healing [23]. Additionally, obstructed microvessels may limit the future potential for collateral blood flow development [23].

**Table 1:** Clinical studies of CMR MO

**Study**	**Method of MO assessment**	**Imaging time post-MI**	**Prevalence of MO**	**Findings**
Positive studies				
Wu et al. [43] N = 44	· Early (yes/no)	10 days	25%	MO predicted MACE at 16 mos. independently of infarct size
Hombach et al. [20] N = 110	· LGE (yes/no)	6 days	46%	Late MO predicted MACE at 7.5 mos., independently of LVEF, LVEDV, infarct size
Bruder et al. [44] N = 67	· EGE	5 days	61%	EGE MO > 0.5% predicted 1-yr MACE independently of LVEF & infarct size
Cochet et al. [45] N = 184	· First-pass LGE	3-7 days	69% (FP)	Late MO predicted 1-yr MACE independently of LVEF & infarct size
			47% (LGE)	
De Waha et al. [46] N = 408	· EGE LGE	3 days	81% (FP)	Late MO (presence/amount) better predicted MACE at 19 mos. with incremental value over LVEF & infarct size
			73% (LGE)	
Negative studies				
Bodi et al. [47] N = 214	· LGE (yes/no)	7 days	31%	Infarct transmural extent & wall motion score independently predicted MACE at 1.5 years.
Larose et al. [48] N = 103	· First-pass	<12 h	Not reported	Infarct size was the strongest predictor of 6 mo. LVEF & 2-yr MACE
		6 months		

**Table 2:** CMR MO and LV remodeling in patients

**Study**	**Method of MO assessment (yes/no for all)**	**Imaging time post-MI**	**Prevalence of MO**	**Findings**
Positive studies				
Wu et al. [43] N = 44	· Early	· 10 days	25%	Early MO predicted LV remodeling
		· 16 months		
Hombach et al. [20] N = 110	· LGE	· 6 days	46%	Late MO, infarct size predicted LV remodeling
		· 7.5 months		
Nijveldt et al. [49] N = 60	· First-pass	· 2-9 days	· 68% (FP	Late MO was the strongest predictor of LV remodeling
	· LGE	· 4 months	· 57% (LGE)	
Orn et al. [30] N = 42	· First-pass	· 2 days	Day 2:	Late MO was associated with the worst LV remodeling indices at all timepoints
	· LGE	· 1 week	· 38% (FP)	
		· 2 months	· 33% (LGE)	
		· 1 year		
			· 1 week:	
			· 34% (FP)	
			· 21% (LGE)	
Weir et al. [50] N = 100 (LVEF < 40%)	· Early	· 4 days	· 69% (Early)	Late MO was associated with the greatest change in LV remodeling indices
	· LGE	· 24 weeks		
			· 56% (LGE)	
Negative studies				
Mather et al. [33] N = 48	· LGE	· 2 days	Day 2 & 1 week	1 week infarct size & LVEF best predicted 3 month infarct size & LVEF
		· 1 week	· 60%	
		· 1 month		
		· 3 months		

An experimental model using myocardial tissue tagging suggests a possible mechanism linking MO to poor infarct remodeling: MO may cause heterogeneity in local myocardial tissue properties across the infarct wall that could adversely affect wall stress and subsequent infarct healing [25]. In a canine model of acute reperfused MI, myocardial deformation was measured at 4–6 h, 48 h, and 10 days post-reperfusion. MO extent, measured both early (4–6 h post-reperfusion) and at 48 h, was a stronger predictor of increases in LV volumes than infarct size. There were demonstrable changes in myocardial deformation up to 48 h post-reperfusion in the infarcted and adjacent myocardium based upon the amount of MO, expressed as a percent of infarct size. Compared to animals with MO comprising <35% of the infarct volume, in those with MO comprising >35% of the infarct volume, infarcted myocardial segments exhibited significantly less stretching in the longitudinal direction; noninfarcted, adjacent regions exhibited reduced radial thickening; and there was a reduction in the first principal strain. This suggests that infarcts with greater amounts of MO are characterized by increased stiffness and reduced myocardial deformation in both the necrotic region and also the adjacent, noninfarcted region. Changes within the infarct region were seen at 4–6 h and preceded the alterations in the adjacent segments, which were not observed until 48 h post-reperfusion. Thus, increased myocardial stiffness and reduced elasticity in the infarcted regions with large amounts of MO could result in increased local wall stress that subsequently limits systolic shortening in adjacent segments, all of which may lead to adverse LV remodeling.

Most of the human studies showing a positive independent association between MO and clinical outcome tended to measure MO within 3–7 days post-reperfusion and utilized the LGE technique for assessment of MO. There have been a few studies that have suggested that MO is not independently predictive of outcome, after controlling for infarct size [47,48] (Table 1). In one of the studies, MO was measured relatively early post-infarct (median of 4.5 h) which may have underestimated the true incidence of MO since MO likely increases in size over the first 24–48 h post-reperfusion. In the other study, the study results may have been influenced by heterogeneity in the times to actual epicardial revascularization which will affect the evolution of MO [51].

### MO and infarct hemorrhage

**Figure 10:**
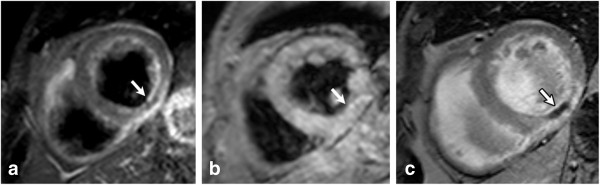
**Shown are short axis images from a 48-year-old gentleman who presented with inferior ST-elevation and received primary percutaneous intervention to the RCA.** CMR on day 2 post MI demonstrated an area of hypointense signal on T2-weighted imaging (Panel **a**), but no signal hypointensity on T2* imaging (Panel **b**). This area was confirmed to represent MO on early gadolinium imaging (Panel **c**). Intramyocardial hemorrhage results in decreased signal intensity on T2* imaging, and the absence of this feature reflects a lack of visible hemorrhage within the area of MO. (Image provided courtesy of Ananth Kidambi, BMBCh; Adam N. Mather, MBBS; and Sven Plein, MD, PhD; Multidisciplinary Cardiovascular Research Centre & Leeds Institute of Genetics, Health, and Therapeutics, University of Leeds, Leeds, United Kingdom.).

As part of the disruption to the microvasculature observed upon reperfusion, large gaps can be seen in the endothelial wall that cause extravasation of red blood cells, i.e. hemorrhage. In experimental models performed as early as the 1980’s, it was observed that any hemorrhage was limited to the region of severe microvascular injury,. lagged behind the no-reflow process and its extent was directly proportional to the duration and severity of the preceding ischemia (with the severity of ischemia determined predominantly by collateral flow) [22,52-58]. Specifically, myocardial hemorrhages occurred when myocardial blood flow during the time of coronary occlusion prior to reperfusion was <21% of control [54]. Macroscopic hemorrhage does not occur in the absence of reperfusion, i.e. in the presence of a persistent coronary occlusion [55] (Figure 10). The extent of hemorrhage has been found to be highly correlated with pathologic infarct size (r = 0.90) and increases in proportion to the occlusion time prior to reperfusion but is independent of thrombolytic therapy (i.e. a lytic state does not increase the amount of hemorrhage seen) [59-61].

**Figure 11:**
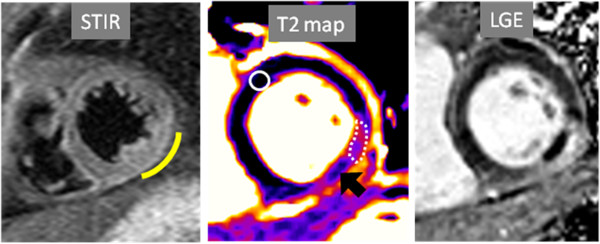
**T2-weighted short tau inversion recovery (STIR), T2 map and late gadolinium enhancement (LGE) images – all obtained in a similar mid-short axis plane – are shown from CMR examination performed in a 53 year-old male who suffered ST-elevation myocardial infarction.** CMR performed 1 day following percutaneous coronary revascularization of a large obtuse marginal coronary artery yielded a calculated LVEF of 55%. STIR imaging suggested increased signal intensity in the inferolateral wall (arc), but does not demonstrate an abnormality within this region. Quantitative T2 mapping shows a region of increased T2 with a hypointense core indicating microvascular obstruction (MO). Mean T2 values in the edematous region (dotted line), MO (black arrow) and remote myocardium (solid circle) were 71, 59 and 48 msecs. 10 min after intravenous gadolinium-based contrast administration, LGE confirms a region of MO in this post-STEMI patient. (Image provided courtesy of Subha V. Raman, MD, MSEE; Davis Heart and Lung Research Institute, The Ohio State University, Columbus, Ohio.).

Hemorrhage can be assessed using T2-weighted and T2* imaging. The appearance of hemorrhage on MRI is based upon the paramagnetic effects of hemoglobin degradation products [63,64]. Initially, hemorrhage may consist of oxyhemoglobin which lacks paramagnetic effects. Subsequently, probably within the first few days after acute MI, oxyhemoglobin denatures into deoxyhemoglobin which does exert paramagnetic effects and will significantly reduce the T2 time. Deoxyhemoglobin is then gradually converted over the following few days into methemoglobin, which is strongly paramagnetic with respect to both T1- and T2-weighted sequences. After ~2 weeks, methemoglobin is converted into hemosiderin which is contained within macrophages and also results in low T2 values. Hence, acutely post-MI, hemorrhage can be visualized on CMR as hypoenhanced regions surrounded by elevated signal intensity representing myocardial edema on T2-weighted imaging and most studies show that hemorrhage is limited to those patients with evidence of MO [26,65-67]. Evaluation of human autopsy specimens in 2 cases showed a close correlation between pathologic hemorrhage and hypoenhanced signal intensity on T2 sequences [68]. However, MVO without hemorrhage can also result in hypoenhanced regions seen on T2-weighted sequences [69] and both can appear as rims of persistent hypoenhancement on LGE [70,71] (Figure 11). A potentially promising approach in differentiating MVO with and without hemorrhage could be the method of direct T2 quantification, which has yielded more robust findings for the detection of myocardial edema compared to conventional T2-weighted short tau inversion recovery [72,73]. Within the infarct core, T2 mapping demonstrates significant reductions in T2 values corresponding to LGE MO (Figure 11). Whether or not such a technique can also differentiate between MO with and without hemorrhage by detecting gradations in reduced T2 values requires further investigation.

**Figure 12:**
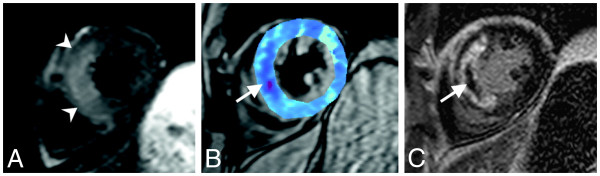
The potential for T2* imaging to improve the differentiation between MO with and without hemorrhage is shown. Panel **A** shows uniform hyperintensity on T2-SPIR (spectrally selective inversion recovery) imaging despite a region of persistent MO on LGE (Panel **C**). In Panel **B**, a small region of decreased T2* is shown, which is much smaller than the MO region, suggesting that much of MO region is in fact non-hemorrhagic and shows normal T2* values. (Reprinted with permission from: O’Regan DP et al. Heart 2010; 96:1885-1891 [78]). See also the imaging vignette from Cannan C et al. JACC: Cardiovascular Imaging 2010; 3(6):665–668 [71].

As opposed to T2 relaxation which depends primarily on spin-spin interactions, T2* decay is caused by a combination of spin-spin relaxation and magnetic field inhomogeneity [74]. Because iron deposits and blood products cause magnetic field inhomogeneities, T2* imaging has been used to measure myocardial iron deposition in the setting of iron overload cardiomyopathies and recently applied to detecting hemorrhage in the setting of acute reperfused MI [75-78]. Since the paramagnetic effects of hemoglobin byproducts on T2* is even stronger than on T2, T2*-weighted sequences should generally exhibit higher sensitivity for hemorrhage [78-80]. In a recent experimental canine model, T2* imaging was compared to pathologic analysis of the infarct region at day 3 post-reperfusion [76]. Gross hemorrhage was limited to those animals with the largest infarct sizes and extents of MVO. Pathologically, all animals with hemorrhage had evidence of MVO. There was high concordance between hemorrhage detected pathologically and that detected by T2* imaging (kappa = 0.96, P < 0.01). The size of the hemorrhagic area was highly correlated with that measured pathologically (r = 0.91, p < 0.01), though CMR overestimated the extent (mean difference of 2.4 ± 1.4%). The amount of hemorrhage was always smaller than the amount of MVO and was contained within it. Use of T2* sequences has the potential to improve the differentiation of MVO alone from MVO with hemorrhage: hemorrhage will be accompanied by reduced, below normal T2* levels whereas MVO alone will not [71] (Figure 12).

The time course over 6 weeks of MO and hemorrhage was further examined recently in a porcine model of acute reperfused myocardial infarction [29]. MO was assessed using EGE and hemorrhage by T2* mapping at 2 days and 1, 2, 4, and 6 weeks post-reperfusion. MO extent was the largest at 2 days with gradual reduction over the ensuing 2 weeks and complete resolution by week 4. T2* values reflecting hemorrhage followed the same temporal course with the lowest T2* levels seen acutely in the hemorrhagic core which gradually normalized to control levels at 4 weeks. Detailed histopathologic analysis was also performed and compared to the CMR findings. At day 2 post-infarct, T2* signal voids corresponded to histopathologic areas of extensive hemorrhage within the infarct. At week 2, histologic sections within the MVO region continued to show obstructed microvessels with necrotic debris and macrophage infiltration. Iron deposition within the MVO region corresponded to T2* signal voids at this time point. By 6 weeks post-MI, the infarct region was characterized by extensive collagen deposition and myocardial fibrosis with no evidence of iron deposition consistent with resolution of the hemorrhage and MVO process. In this study, only infarcts with hemorrhage were studied.

Subsequent clinical studies have supported these experimental findings. In patients, the extent of late MO has been found to be highly correlated with the size of the hemorrhage area (r^2^ = 0.87, p < 0.001) and interestingly, the correlation with early MO was less robust (r^2^ = 0.30, p < 0.003) [78]. Several other studies have shown that the presence of hemorrhage is a strong predictor of adverse LV remodeling and MACE (Table 3). Thus, the data suggest that hemorrhage reflects more severe, and possibly irreversible, persistent, “structural” rather than potentially reversible, “functional” microvascular injury and its resolution parallels that of anatomic MVO as infarct healing ensues. It may thus be a more specific marker of adverse events because it reflects irreversible, structural injury to the microvasculature, but this requires further investigation and confirmation.

**Table 3:** Clinical Studies of Myocardial Hemorrhage

**Study**	**CMR methods**	**Imaging time post-MI**	**Prevalence of MO & hemorrhage (H)**	**Findings**
Ganame et al. [66] N = 98	· EGE (MO)	· 1 week	64% (MO)	Presence of hemorrhage & infarct size were the strongest predictors of LV remodeling
	· T2W (H)	· 4 months	24% (H)	
Mather et al. [33,81] N = 48	· EGE (MO)	· Day 2	63% (MO)	Presence of hemorrhage, LVEF & infarct size were the best predictors of adverse remodeling
	· T2W & T2* (H)	· 3 months	25% (H)	
Eitel et al. [67] N = 346	· LGE (MO)	· Day 3	70% (MO)	Presence of hemorrhage & LVEF were the best predictors of 6 month MACE
	· T2W (H)			
			35% (H)	

### Clinical trial applications

MO is increasingly incorporated into clinical trial methodology as a surrogate clinical outcome in studies investigating adjunctive reperfusion agents and strategies, particularly when the treatment has the potential to directly impact microvascular function [82-92]. On the web-based clinical trial registry, http://www.clinicaltrials.gov, there are currently 25 open studies in which microvascular obstruction is an endpoint and there are an additional 22 closed studies ( http://clinicaltrials.gov/ct2/results?term=microvascular+obstruction). In doing so, early signals of detrimental effects may be detected before larger scale trials are embarked upon. An example of this was observed in a 51 patient randomized study of erythropoietin (EPO), in which there was a doubling of MO incidence in the treated group, associated with increased LV volumes acutely post-MI [83]. Although the mechanisms for this are not definitively known, the contribution of increased platelet reactivity and the prothrombotic effects of EPO provide a plausible explanation for the observed increase in MO. Notably, there was a nonsignificant trend toward an increased infarct size in the treated group in this study. In a subsequent trial in which a single high dose of EPO was administered to 110 patients, there was no change in infarct size at 3 months but MO incidence was significantly decreased acutely [93]. The authors postulated that the higher dose and earlier delivery of EPO may have accounted for the differences in MO rates. Nonetheless, both of these studies highlight the potential for MO evaluation to provide better study power as an endpoint compared to infarct size.

A potentially valuable application is in the evaluation of stem cell therapy for acute MI, particularly when such cells are delivered via the intracoronary route. Many of such published human studies have used CMR to predominantly assess LVEF and LV volumes with an increasing number also assessing infarct size [94-99] and MO [96,100-103]. As larger efficacy trials are being conducted and planned, incorporating systematic MO assessment may help in identifying which patients may derive the most benefit (i.e. larger amounts of MO could theoretically impair stem cell engraftment); optimizing the timing of cell delivery in relation to MO temporal evolution; and understanding possible pathophysiologic benefits of stem cells (i.e. is there neovascularization or restoration of microvascular function or are there detrimental effects on microvascular perfusion due to cellular plugging of microvessels [104]).

## Ongoing controversies and future directions

Currently, the CMR assessment of MO is limited by the lack of standardization with regard to when temporally MO is measured, both with respect to the time following contrast administration and the time post-reperfusion. There is likely to be marked fluidity of MO presence and extent as infarct healing ensues and as a function of inter-patient variability in the degree/reversibility of microvascular dysfunction. FP perfusion and EGE/LGE techniques, particularly when measured at only one timepoint post-reperfusion, may overestimate the extent of structural, anatomic MO since other reversible factors may affect gadolinium contrast kinetics, such as extrinsic compression of the microvessels or vasoconstriction, which may resolve much more quickly than anatomic MO. Whether or not the assessment of myocardial hemorrhage improves the diagnostic utility of MO remains undetermined, as does the optimal method to detect hemorrhage. A certain amount of hemorrhage is seen in all reperfused infarcts and likely reflects the severity of the ischemic microvascular injury. MO by itself, without hemorrhage, may limit the effective amount of tissue water because of the associated cellular debris and obstructed capillary flow thereby reducing the supply of protons within the infarct volume and thus lead to areas of low T2. Newer techniques such as T2* sequences could theoretically be more sensitive and specific for detecting *severe* anatomic microvascular injury, specifically, the consequences of endothelial injury and subsequent extravasation of blood, by specifically measuring hemoglobin byproducts. However, larger studies are needed to examine the comparative diagnostic performance of various measures of CMR microvascular dysfunction in predicting adverse LV remodeling and clinical outcome. Consideration should be given to examining MO and infarct hemorrhage at various time points within the first 7–10 days post-MI to perhaps distinguish between functional from anatomic MO,, particularly in clinical treatment trials of adjunctive reperfusion agents or strategies.

Our knowledge regarding how CMR measures of MO directly compare with indices of microvascular dysfunction assessed by other imaging modalities remains incomplete but an increasingly number of studies are addressing this question. CMR has been compared with acute electrocardiographic ST segment resolution [49,105-108], angiographic measures [49,107,109-111], myocardial contrast echo [12,38] and recently, multidetector cardiac CT [112-114]. The methods generally correlate fairly well with one another in terms of diagnostic utility for MO. However, there is no consensus regarding the “best” method to assess MO in terms of pathophysiology, diagnostic performance, and prognostic implications. Given that angiographic indices and electrocardiographic ST segment assessment can be performed acutely during occlusion/reperfusion, they may in fact be complementary to the CMR indices, which are generally assessed later in the post-MI period. Further investigation is warranted as to the comparative roles and diagnostic and prognostic value of the different imaging methods to assess MO, particularly since many are increasingly being used as surrogate clinical trial endpoints.

## Conclusions

In summary, microvascular obstruction and the related phenomenon of myocardial hemorrhage, occur commonly following reperfusion for acute MI and can be assessed using various CMR techniques, including first pass perfusion, early and late gadolinium enhancement, and recently, T2 and T2* techniques. Both experimental and human studies support the association between the increasing incidence and extent of MO and worse LV remodeling, likely as a result of the adverse consequences of increased myocardial stiffness and reduced deformation in both the infarct and adjacent regions. The adverse effect on the infarct healing process in turn may explain the worse prognosis observed with MO. However, there has not yet been definitive proof that MO can be modified by any intervention and in so doing, results in improved outcome. This is in part due to the lack of a standardized approach to assessing MO, which will need to be established, both with respect to the optimal CMR protocol as well as the timing of evaluation post-MI. Thus, while further validation and prognostic studies with larger sample sizes are needed to define its role both in clinical risk stratification and as a research tool, CMR MO assessment will be critical to advancing the treatment of patients with acute MI.

## Competing interests

The author declares that she has no competing interests.
